# Comparison of Home-Blenderized Formula and Commercial Enteral Formulas for Gastrostomy Tube-Fed Children: A Retrospective, Prospective Cohort Study

**DOI:** 10.7759/cureus.37944

**Published:** 2023-04-21

**Authors:** Sebastian Shrager, Ayoola Adigun, Sonia Motolongo, Cristhiane S Santos, Patricia Rowe-King, Debora Duro

**Affiliations:** 1 Pediatrics, Salah Foundation Children’s Hospital at Broward Health Medical Center, Fort Lauderdale, USA; 2 Pediatric Gastroenterology, Salah Foundation Children’s Hospital at Broward Health Medical Center, Fort Lauderdale, USA; 3 Pediatrics, Nova Southeastern University Dr. Kiran C. Patel College of Osteopathic Medicine, Fort Lauderdale, USA; 4 Pediatrics, Florida International University, Herbert Wertheim College of Medicine, Miami, USA

**Keywords:** pediatrics, clinical outcomes, enteral feeding, blenderized formulas, blenderized tube feeding

## Abstract

Background

Blenderized gastrostomy tube feedings (BGTFs) consist of pureed table foods and liquids that are administered as enteral tube feedings. Compared to commercial enteral formulas (CEFs), BGTF has been shown to have fewer side effects. Despite these results, apprehensions have been raised about microbial contamination, nutritional deficiencies or surplus, risk of gastrostomy tube (GT) blockages, and lack of consistency in clinical outcomes. The goal of this retrospective, prospective, 18-month-long study is to report the clinical and nutritional outcomes of GT-dependent pediatric patients who attended a multidisciplinary feeding clinic.

Methodology

After Institutional Review Board (IRB) approval and consent were obtained, 25 children who were receiving tube feeding via G were enrolled in a retrospective, prospective, observational, cohort study from August 2019 to February 2021. A multidisciplinary team was formed, and multivariate logistic regression was performed comparing subjects on BGTF versus CEF, per os diet versus nil per os, CEF versus homemade blenderized tube feeding (HBTF) versus blenderized tube feeding (BTF), and how they compared at the beginning and end of the study.

Results

The mean age of the patients was 4.4 years (SD ±2.2). Gastroesophageal reflux disease (GERD) and short bowel syndrome (SBS) were the most common comorbid gastrointestinal (GI) conditions. Of the 25 patients enrolled in the study, seven were initially on BGTF, while 14 ended the study on BGTF. There were no statistically significant differences in malnutrition status, feeding intolerance, emergency room visits, hospitalizations, and GT blockages between all different comparison groups when comparing between the CEF versus HBTF versus commercial blenderized tube feeding (CBTF) groups. Of the patients who were in the BGTF group, there was a resolution of vitamin A deficiency, vitamin D deficiency, and anemia (n = 1). In total, two patients had resolved vitamin deficiencies, namely, vitamins A and D.

Conclusions

When comparing BGTF and CEF, there was no statistically significant difference in outcomes. This study suggests that BGTF is at least equivalent to CEF in clinical outcomes, meaning BGTF should be considered standard nutrition for GT-dependent patients.

## Introduction

Enteral feeding has been present in many different facets throughout the course of human history, starting with the Greeks and Egyptians providing nutrition through rectal clay pipes, then in 1598 Capivacceus used hollow tubes with a bladder via the esophagus, followed by Von Helmont’s 17th-century leather tubing, Hunter’s 18th-century whalebone-encased eel skin orogastric tube, with rubber tubing not arriving until the 19th century [1]. Nasogastric tube feeds were a viable source of nutrient delivery in the mid-20th century [2] with the first gastrostomy tube (GT) being placed in a child in the 1980s [3]. The issue today is no longer about the mode of delivery, but rather that of quality of nutrient content.

Conventional commercial enteral formulas (CEFs) were developed in the 1970s [4], which replaced institutionally prepared feeds because of their known nutrient content, ease of use, and lack of possible microbial contamination [5]. Although popular, the CEF-based diet lacks natural fiber while also being high in processed carbohydrates and saturated fat and packed with preservatives, which may be linked to pulmonary, cardiovascular, and inflammatory disease [6,7]. This is not only problematic for a healthy population but can be particularly detrimental to the growing pediatric population that relies on enteral feeds [6], especially those with complex chronic conditions [7].

Blenderized gastrostomy tube feedings (BGTFs) are defined as the use of blenderized food and liquids given directly via the feeding tube [8]. These table foods consist of vegetable, fruits, meats, and legumes that normalize mealtime, allows the patient and their caregiver to contribute toward meal preparation [9], and are perceived to be healthier and more natural with the option of using organic, GMO-free, whole foods [10]. With BGTF, we can address food allergies and sensitivities and specify the diet based on nutritional preferences such as lactose intolerance, soy intolerance, ketogenic diet, vegetarian, and vegan diets [9]. Compared to CEF, BGTF has been shown to decrease the side effects of gagging, vomiting, constipation, retching, eczema, and oral feeding aversion; improve volume tolerance; and help increase the number of patients transitioning from enteral to oral feedings [9].

Despite these results, apprehensions have been raised about microbial contamination, nutritional deficiencies or surplus, risk of GT blockages, and lack of consistency in clinical outcomes. Therefore, the purpose of this retrospective, prospective, single-center, cohort study is to determine the nutritional and clinical outcomes of pediatric patients aged 1-18 years, who rely on GT feeding for enteral support and to report our preliminary clinical and nutritional outcomes. The American Society of Parenteral and Enteral Nutrition (ASPEN) defines enteral support as involving the provision of adequate nutrition and fluids through the intestinal tract to enhance appropriate growth and hydration with or without parenteral support or nutrition [11].

The goal of this 18-month-long study is to report the clinical and nutritional outcomes of GT-dependent pediatric patients who attended this multidisciplinary feeding clinic.

Our choice of children who are G-tube dependent as the study population is that the administration of commercial enteral formulas (CEF) via gastrostomy may induce adverse gastrointestinal (GI) symptoms such as vomiting, lack of appetite, and gagging and has also been implicated in reducing the diversity of microbial species in the gut microbiome. Our hypothesis is that children who receive BGTF will have better nutritional and clinical outcomes compared to those on CEF.

This article was previously presented as an abstract at the annual meeting of the American Society of Parenteral Nutrition (ASPEN) Nutrition Science and Practice Conference on March 2021.

## Materials and methods

Study design and cohort

After obtaining Institutional Review Board (IRB) approval from Broward Health Medical Center and consent, 25 children who were receiving GT feedings and being followed at an ambulatory outpatient clinic at Salah Foundation Children’s Hospital were enrolled in a retrospective, prospective, observational, cohort study from August 2019 to February 2021. The justification of our study design is that the use of a cohort study design helps us better measure and control the outcomes and predictor and confounding variables. Previous studies by Chandrasekar et al. [12], Jolfaie et al. [13], Papakostas et al. [14], Orel et al. [15], and Vieira et al. [16] all used such a study design.

Patients were seen by a multidisciplinary team including a pediatric gastroenterologist, three dietitians, a speech therapist, and pediatric residents. The GT feeding clinic is once a month and patients are seen every two to four months based on clinical needs.

Inclusion criteria for the study were (i) patients receiving nutrition via a GT (≥12 French), (ii) patients between the ages of one month and 18 years, and (iii) patients attending the clinic at least twice during the course of the study. Children who were fed via nasogastric or jejunal tubes were excluded from the study.

At each visit, registered dieticians educated the guardians/caregivers of the patients about GT feedings. Nutrition assessments were performed, and nutritional adjustments were made to the feeding regimen to ensure adequate nutritional intake. At each visit, data were collected on anthropometrics, type of GT feeds, nutritional composition, number of emergency room (ER) visits and hospitalizations, malnutrition status, feeding intolerance severity, feeding intolerance frequency, and frequency of feeding tube blockages. Lab work including serum chemistry and vitamin/mineral levels collected once a year. The types of GT feeds were defined as CEF and BGTF. BGTF was further subdivided into commercial BTF (CBTF) and homemade BTF (HBTF). HBTFs contained 1,000-1,800 kcal/day with macronutrients being broken down as follows: 50-55% carbohydrates, 30-35% fat, and 12-15% protein.

Malnutrition was classified as mild if the body mass index (BMI) z-score was between -1 and -2, moderate if between -2 and -3, and severe if greater than -3. Malnutrition was considered acute if lasting for <3 months and chronic if lasting for ≥3 months. Feeding intolerance was based on the following symptoms: gagging, vomiting, constipation, retching, eczema, or oral feeding aversion. Patients were classified as mild if they had one or fewer of these symptoms, moderate if they had two or three symptoms, and severe if they reported more than three adverse symptoms. Feeding intolerance frequency was defined as mild if the patient had these symptoms for a month or less, moderate if weekly, and severe if daily. Feeding tube blockage frequency was defined as mild if the patient experienced tube blockages monthly, moderate was defined as weekly, and severe was defined as daily.

Patients were stratified into multiple groups including CEF versus BGTF and CEF versus HBTF versus CBTF. A patient was part of the CEF group if the only form of GT feeds that the patient was receiving at the time was CEF. A patient was part of the BGTF group if they were receiving CBTF or HBTF at the time. A patient was in the HBTF group if a patient was receiving homemade blenderized GT feeds as part of their nutrition at the time of evaluation. A patient was considered part of the CBTF group if the patient was receiving commercial blenderized GT feeds and was not receiving any homemade blenderized GT feeds as part of the nutrition at the time of evaluation. The study also compared patients who had a per os (PO) diet versus a nil per os (NPO) diet.

Statistical analysis

Descriptive statistics were performed on all patient characteristics, comorbid conditions, and anthropometric measurements for all 25 enrolled pediatric patients in the study. Results are reported as frequency and percentage (%) for categorical variables, while continuous variables are summarized as median and standard deviation (±SD).

Furthermore, the study used multivariate logistic regression to examine the association between the outcome variables and predictor variables (CEF versus BGTF, PO intake versus NPO intake, CEF versus HBTF versus CBTF). The outcome variables included weight percentile, BMI percentile, ER visits, hospitalizations, malnutrition severity, feeding intolerance severity, feeding intolerance frequency, and frequency of feeding tube blockages. Results were interpreted as adjusted odds ratio (AOR) and 95% confidence intervals (CIs). Statistical significance was determined by a two-sided p-value <0.05. All analyses were performed using STATA version 16 for Mac (StataCorp LLC, College Station, TX, USA).

## Results

Patient characteristics

In total, 11 (44%) patients were male and 14 (66%) were female, as shown in Table 1. The average age was four years and eight months (±27 months.) The most common GI diagnoses were gastroesophageal reflux disease (GERD) in 10 (40%) patients and short bowel syndrome (SBS) in nine (36%) patients, small intestinal bowel overgrowth (SIBO) in eight (32%) patients, and total parenteral nutrition (TPN) dependence in eight (32%) patients. The average BMI (z-score) was 0.6 (1.6). Seven (28%) patients received BGTF, and 18 (72%) patients received CEF only at the beginning of the study. Fifteen (60%) patients received BGTF, and 10 (40%) patients received CEF at the end of the study. There were seven (28%) patients who stayed on CEF throughout the course of the study, 10 (40%) patients who stayed on BGTF throughout the course of the study, and eight (32%) patients who transitioned from CEF to BGTF. A total of seven (28%) patients were in the HBTF group and seven (28%) patients in the CBTF group at the end of the study.

**Table 1:** Anthropometric data of study participants. *: CEF: if the patient received commercial enteral formula only; #: BGTF: if the patient received HBTF or CBTF; ^1^: CEF→CEF: if the patient was receiving CEF on their first visit and was receiving CEF on their last visit to the clinic; ^2^: BGTF→BGTF: if the patient was receiving BGTF on their first visit and was receiving BGTF on their last visit to the clinic; ^3^: CEF→BGTF: if the patient was receiving CEF on their first visit and was receiving BGTF on their last visit to the clinic. CEF: commercial enteral formula; BGTF: blenderized gastronomy tube feed; HBTF: homemade blenderized tube feed; CBTF: commercial blenderized tube feed

Variables	Number (%), mean (±SD)
Gender	
Male	11 (44)
Female	14 (66)
Age	4 years and 8 months (±27 months)
Gastrointestinal diagnosis	
Gastroesophageal reflux	10
Short bowel syndrome	9
Small intestinal bowel overgrowth	8
Total parenteral nutrition dependence	8
Necrotizing enterocolitis	5
Oral aversion	3
Chronic constipation	3
Cleft palate	3
Feeding intolerance	3
Chronic diarrhea	2
Esophageal atresia s/p repair	1
Intestinal malrotation	1
Jejunal stricture	1
Milk protein allergy	1
Anthropometrics	
Weight (kg)	15.6 (4.6)
Weight (z-score)	-1.5 (1.8)
Height (cm)	97.4 (14.9)
Height (z-score)	-1.7 (1.4)
Body mass index (kg/m²)	15.6 (2.3)
Body mass index (z-score)	-0.6 (1.6)
Group	
¹ CEF→CEF*	10
² BGTF→BGTF^#^	7
³ CEF→BGTF	8

Comparison between groups

When comparing the data between the BGTF and CEF groups at the beginning and end of the study, there was no statistically significant difference in the anthropometric data such as BMI, malnutrition status, weight, or height, as shown in Table 2. For example, patients at the end of the study were 1.6 times more likely to be hospitalized (p = 0.676) and 0.67 times less likely to visit the ER (p = 0.300). Patients were also 0.37 times less likely to have mild feeding intolerance (p = 0.651) and 0.58 times less likely to have moderate feeding intolerance (p = 0.549) at the end of the study.

**Table 2:** Multiple logistic regression showing patient outcome measures compared with blenderized gastrotomy tube feedings versus commercial enteral formulas.

Predictor variable	Beginning of the study		At the end of the study	
	Blenderized gastrotomy tube feedings (N = 7), odds ratio (95% CI)	P-value	Blenderized gastrotomy tube feedings (N = 15), odds ratio (95% CI)	P-value
Weight (%)	1.00 (0.96–1.04)	0.969	0.99 (0.96–1.02)	0.576
Body mass index (%)	1.00 (0.98–1.03)	0.486	0.99 (0.96–1.02)	0.548
Height (%)	0.99 (0.96–1.04)	0.992	0.98 (0.95–1.02)	0.376
Malnutrition status (reference: none)				
Mild	0.67 (0.05–7.85)	0.747	0.45 (0.03–5.84)	0.542
Moderate	1.00 (--)	--	1.00 (--)	--
Severe	1.00 (--)	--	0.90 (0.05–16.59)	0.944
Hospital Admissions	1.40 (0.19–10.14)	0.739	1.63 (0.16–16.34)	0.676
Emergency room visits	0.33 (0.03–3.43)	0.356	0.23 (0.01–3.64)	0.300
Feeding intolerance severity (reference: none)				
Mild	2.27 (0.20–24.88)	0.501	0.63 (0.08–4.59)	0.651
Moderate	2.50 (0.09–62.60)	0.577	0.42 (0.02–7.15)	0.549
Feeding intolerance frequency (reference: none)				
Rare	3.00 (0.23–37.67)	0.395	0.24 (0.01–3.30)	0.288
Sometimes	1.00 (--)	0.326	1.00 (--)	--
Often	3.60 (0.27–46.36)	0.097	1.00 (--)	--
Feeding tube blockages (reference: none)				
All the time	1.00 (--)	--	1.00 (--)	--

There was also no statistically significant difference in the patient outcomes when comparing across clinical outcomes, including ER visits, hospitalizations, feeding intolerance severity, feeding intolerance frequency, and frequency of feeding tube blockages, suggesting that BGTF is at least equivalent to CEF. When comparing CGT-BGTF (n = 10), BGTF-BGTF (n = 7), and CEF-BGTF (n = 8) again, there was no statistically significant difference between the groups. The most common feeding intolerance was oral feeding aversion across all three groups. When comparing patients with PO versus NPO, there was a statistically significant difference in BMI of 0.7 (0.54-0.91) with a p-value of 0.009, as shown in Table 3. Patients on PO were less likely to have at least one ER visit (AOR = 0.34, 95% CI = 0.10-1.14) and two ER visits (AOR = 0.40, 95% CI = 0.02-6.71).

**Table 3:** Multivariate logistic regression showing patient outcome measures comparing those with per os intake versus those without.

Predictor variable	PO intake (N = 58), odds ratio (95% CI)	P-value
Weight (cm)	0.98 (0.88–1.08)	0.703
Body mass index (kg/m^2^)	0.70 (0.54–0.91)	0.009*
Malnutrition status (reference: none)		
Mild	5.40 (0.63–45.93)	0.122
Moderate	3.38 (0.37–30.67)	0.279
Severe	5.40 (0.63–45.94)	0.122
Number of hospital admissions (reference: 0)		
1	1.22 (0.38–3.90)	0.729
2	1.00 (--)	--
Number of emergency room visits (reference: 0)		
1	0.34 (0.10–1.14)	0.082
2	1.00 (--)	--
3	0.40 (0.02–6.71)	0.524
Feeding intolerance severity (reference: none)		
Mild	1.55 (0.56–4.24)	0.394
Moderate	0.64 (0.17–2.39)	0.509
Feeding intolerance frequency (reference: none)		
Rare	0.71 (0.23–2.15)	0.552
Sometimes	1.10 (0.08–13.54)	0.941
Often	2.10 (0.66–6.73)	0.208
Feeding tube blockages (reference: none)		
All the time	1.00 (--)	--

Table 4 shows that patients on CEF were 2.1 times more likely to have hospital visits (AOR = 2.17, p = 0.449), 1.6 times more likely to have severe malnutrition (AOR = 1.60, p = 0.758), and 0.2 times less likely to have mild feeding intolerance (AOR = 1.07, p = 0.949) compared to those on CBTF, although the results were not statistically significant.

**Table 4:** Multivariate logistic regression showing patient outcome measures comparing those with commercial blenderized tube feeds versus commercial enteral formulas at the end of the study.

Predictor variable	Commercial enteral formulas (N = 7), odds ratio (95% CI)	P-value*
Weight (%)	1.02 (0.98–1.05)	0.236
Body mass index (%)	1.00 (0.98–1.04)	0.569
Height (%)	0.99 (0.96–1.03)	0.967
Hospital visits	2.17 (0.29–16.41)	0.449
Emergency visits	1.00 (--)	--
Malnutrition status (reference: none)		
Mild	0.80 (0.05–11.29)	0.869
Moderate	--	--
Severe	1.60 (0.08–31.77)	0.758
Feeding intolerance severity (reference: none)		
Mild	1.07 (0.14–7.82)	0.949
Moderate	1.00 (--)	--
Feeding intolerance frequency (reference: none)		
Rare	0.44 (0.03–6.70)	0.558
Sometimes	1.00 (--)	--
Often	1.00 (0.12–8.30)	1.000
Feeding tube blockages (reference: none)		
All the time	1.00 (--)	--

Table 5 shows that patients on CBTF were 0.55 times less likely to have mild feeding intolerance (AOR = 0.45, p = 0.602) compared to those on CEF. When comparing CEF (n = 7) versus CBTF (n = 8) versus HBTF (n = 7), there was no statistically significant difference between the groups, as shown in Table 5 and Table 6.

**Table 5:** Multivariate logistic regression showing patient outcome measures comparing those with commercial blenderized tube feeds versus commercial enteral formulas at the end of the study.

Predictor variable	Commercial blenderized tube feeds (N = 8), odds ratio (95% CI)	P-value*
Weight	1.03 (0.97–1.08)	0.292
Body mass index (%)	1.01 (0.97–1.05)	0.472
Height (%)	1.01 (0.96–1.06)	0.589
Hospital visits	1.00 (--)	--
Emergency room visits	1.00 (--)	--
Malnutrition status (reference: none)		
Mild	1.00 (--)	--
Moderate	1.00 (--)	--
Severe	1.00 (--)	--
Feeding intolerance severity (reference: none)		
Mild	0.45 (0.02–8.82)	0.602
Moderate	1.00 (--)	--
Feeding intolerance frequency (reference: none)		
Rare	1.00 (--)	--
Sometimes	1.00 (--)	--
Often	1.20 (0.06–24.47)	0.906
Feeding tube blockages (reference: none)		
All the time	1.00 (--)	--

**Table 6:** Multivariate logistic regression showing patient outcome measures comparing those with home blenderized tube feeds versus commercial enteral formulas at the end of the study.

Predictor variable	Home blenderized tube feeds (N = 7), odds ratio (95% CI)	P-value*
Weight (%)	0.97 (0.93–1.03)	0.433
Body mass index (%)	0.99 (0.95–1.02)	0.490
Height	0.96 (0.91–1.03)	0.292
Hospital visits	1.00 (--)	--
Emergency room visits	0.75 (0.05–10.23)	0.829
Malnutrition status (reference: none)		
Mild	1.00 (--)	--
Moderate	1.00 (--)	--
Severe	1.00 (--)	--
Feeding intolerance severity (reference: none)		
Mild	0.20 (0.01–2.57)	0.217
Moderate	1.00 (0.09–11.02)	1.000
Feeding intolerance frequency (reference: none)		
Rare	0.33 (0.02–4.73)	0.417
Sometimes	1.00 (--)	--
Often	0.25 (0.02–3.34)	0.295
Feeding tube blockages (reference: none)		
All the time	1.00 (--)	--

The results in Table 6 show that patients on CEF were 0.25 times less likely to have ER visits (AOR = 0.75, p = 0.829), 0.8 times less likely to have mild feeding intolerance (AOR = 0.20, p = 0.217), and 0.67 less likely to have rare feeding intolerance frequency (AOR = 0.33, p = 0.417) compared to those on HBTF.

Clinical and nutritional outcomes

From a PO standpoint, one patient in the BGTF-BGTF group went off TPN, and one patient in the CEF-CEF and CEF-BGTF group went from NPO to PO. From an anemia standpoint, one patient in the CEF-CEF group became anemic, one stayed anemic, and one patient’s anemia in the BGTF-BGTF group resolved. From a vitamin A deficiency standpoint, one patient in the CEF-CEF group developed vitamin A deficiency, and one patient in the CEF-BGTF group had their vitamin A deficiency resolved.

From a vitamin D standpoint, one patient in the CEF-BGTF group became vitamin D deficient, and one patient’s vitamin D deficiency resolved. From a phosphorus standpoint, one patient in the CEF-CEF group remained deficient and two patients in the CEF-BGTF group developed hypophosphatemia. One patient in the CEF-CEF group developed zinc deficiency.

## Discussion

Our study is a retrospective, prospective study examining pediatric patients who are GT-dependent. The study attempted to determine the nutrition and clinical outcomes between different GT-dependent patients receiving commercial, blenderized, or mixed feeding regimens or. Major concerns about blenderized tube feedings in the literature include food safety, ER visits, hospitalizations, malnutrition status, feeding intolerance, and GT blockages. Consistent with previous studies, our results show that BGTF can decrease GI symptoms [17,18].

Our study did not show any statistically significant improvement in the BGTF group in comparison to the CEF group. This is clinically significant as our study suggests that BGTF is equivalent to CEF in this cohort of patients. When the data were further examined to compare CEF versus HBTF versus CBTF, we did not find any statistically significant difference. BGTF is made from table foods in which recipes can be individualized to meet all nutritional needs of children or adults. This is particularly relevant today, as there is an increasing population of medically complex children living in the United States, and therefore, an increasing prevalence and incidence of long-term GT feedings [19-21]. Additionally, this is one of the first studies to compare HBTF and CBTF; therefore, we recommend further research in this area involving a larger cohort of patients.

We decided to investigate the differences between commercial, blenderized, and mixed feeding regimens because other studies such as the Blenderized Enteral Nutrition Diet (BLEND) study conducted by the Hospital for Sick Children, Toronto to determine feeding tolerance and assess parental perspectives of this feeding approach showed that the implementation of BGTF is associated with improved clinical outcomes and increased intestinal bacterial diversity [22].

Looking at clinical outcomes, especially in the CEF-BGTF group, where patients were initially on CEF and then transitioned to BGTF, our study showed clinical improvements. Specifically, one patient went from NPO to PO and one patient demonstrated resolution of vitamin A and D deficiency. Moreover, in the comparison group of patients who stayed on the commercial formula for the duration of the study, in the CEF-CEF group, there was one patient who became anemic, one patient each who became vitamin A and zinc deficient, and one patient each who stayed anemic and developed hypophosphatemia. Although we cannot conclude a causal relationship, this pilot retrospective cohort of patients suggests that there is more opportunity for research in the area which involves expanding the sample size and making the results generalizable.

Limitations

Our study has some limitations which include the length of patient follow-up, which did not allow for the determination of long-term outcomes. Ideally, the patients would have been followed over an extended period of time to show differences in the group and allow for the different feeding regimens to take effect. Second, the size of the cohorts, 25 patients total, was not large enough to give the study significant power. When comparing small cohorts such as these, only obvious differences with be noted upon statistical analysis. Third, 46% (n = 7) of patients in the BGTF groups had diets supplemented with CEF. Fourth, this is an observational study, which is not as rigorous as a double-blind, randomized controlled trial. Finally, the COVID-19 pandemic limited the ability to obtain anthropometric data, yearly labs, and, most significantly, provide in-person high-quality dietary GT education to patients.

## Conclusions

Given the growing interest in BGTF for enterally fed children, it is important to investigate the effect of BTF on symptoms, quality of life, growth, nutrition, hospital admissions, ER visits, and feeding intolerance. This study demonstrates that BGTF use is associated with reduced GI symptoms associated with GT feeding. Despite being nutritionally superior to commercial formulas in caloric and micronutrient content, careful dietetic monitoring is required to ensure anthropometric goals are met on BGTF. While larger, prospective studies are needed, this study provides a basis for investigating if a combination of BGTF and CEF may help reduce symptoms, while supporting growth in the ever-growing population of medically complex children in the United States. Even though better-designed studies are necessary to further explore and validate the findings of this retrospective cohort study, practitioners may use a patient-centered approach to discuss the advantages and disadvantages of BGTF based on the evidence in the published literature.

There is a need for further research into BGTF as this early study shows. HBTF and CBTF are so far safe and have shown, at least in our study, to be equivalent to CEF and to have some clinical advantages. A double-blind study with a larger cohort over a more extended period of time would help examine the benefits of blenderized tube feedings. Prospective multicenter studies are needed to further understand the mechanisms by which BGTF can help reduce adverse symptoms associated with pediatric intestinal failure, microbial contamination, and nutritional deficiencies or surplus.
